# A New Quackery

**Published:** 1852-02

**Authors:** 


*A New Quackery.*—In Naumberg a man named Mahner is preaching the necessity of a new regeneration, not in the spiritual but physical sense. He warns a sickly race that it must return to the lost state of "primitive health," or *Ungesundheit*, as the means of more fully enjoying life and attaining a patriarchal old age. It is to be secured by a diet of bread and water, going barefoot, and letting the hair and beard grow; in short, making a nearer approach to man's original state in costume than the decencies or prejudices of modern society will altogether permit. On this topic he has been lecturing to a chosen few, but his doctrines do not seem to take, bread and water not being very tempting, even with four score years promised as the prize of self-denial. It is also said that the apostle does not fully act up to his own precepts, preferring a well-spread table and every variety of wine or beer to the pure element; but this may be a calumny of the hotel-keepers. The German journals are perpetually turning up some eccentricity of this sort; to repair or preserve health the oddest "cures" are resorted to. A section of the public seems determined to escape the hands of the faculty, and die by some irregular process, rather than with the aid of medicine. In a single advertising sheet could recently be counted up a water cure, a grape cure, a milk cure, and a hunger cure; to these must be added another just getting into vogue—some of the former having had their day—it is the cure by muscular exercise, by which cripples from rheumatism are put through a course of gymnastics, and dancing is prescribed for the gout. These, however, are all merely cures or remedies; the "primitive health" theory is founded on a system of living which would literally make "man's life as cheap as beasts'," without much prolonging it. As far as hair and beard go, many of the Berliners are meeting the Naumberg preacher more than half way.—*Ibid.*

